# Supplementary material for: Diversity of RNA Viruses and Circular Viroid-like Elements in Heterobasidion spp. in Near-Natural Forests of Bosnia and Herzegovina
Source: Viruses. 2025 Aug 20;17(8):1144. doi: 10.3390/v17081144 (PMC12390675; doi:10.3390/v17081144)
Supplement: Supplementary file 1 [file viruses-17-01144-s001.zip › viruses-3736338-supplementary.pdf]

Supplementary materials

Table S1. Information of *Heterobasidion* isolates studied.

Isolate no.	Species	Host	Substrate[a]	Latitude N	Longitude E	Collection date	Collector	RNA pool
2118	*H. abietinum*	*Picea abies* OR *Abies alba*	stump	44.474509	16.486317	5.5.2022	L. B. Dálya	ABI
2119	*H. abietinum*	*Picea abies* OR *Abies alba*	stump	44.475479	16.490362	5.5.2022	L. B. Dálya	ABI
2120	*H. annosum*	*Abies alba*	stump	44.488593	16.486055	5.5.2022	L. B. Dálya	2120[b]
2121	*H. parviporum*	*Picea abies* OR *Abies alba*	stump	44.597631	16.512048	9.5.2022	L. B. Dálya	PAR
2122	*H. parviporum*	*Picea abies*	root	44.597560	16.512703	9.5.2022	Z. Stanivuković	PAR
2123	*H. parviporum*	*Picea abies* OR *Abies alba*	stump	44.597338	16.512693	9.5.2022	L. B. Dálya	PAR
2124	*H. abietinum*	*Abies alba*	stump	45.012327	16.910220	17.5.2022	L. B. Dálya	ABI
2125	*H. abietinum*	*Abies alba*	stump	45.011899	16.907795	17.5.2022	L. B. Dálya	ABI
2126	*H. abietinum*	*Abies alba*	stump	45.011773	16.906346	17.5.2022	L. B. Dálya	ABI
2127	*H. abietinum*	*Abies alba*	stump	45.011793	16.901961	17.5.2022	L. B. Dálya	ABI
2140	*H. abietinum*	*Abies alba*	stump	43.349058	18.614631	14.11.2022	L. B. Dálya	BIH
2141	*H. abietinum*	*Abies alba*	log	43.349284	18.613683	14.11.2022	L. B. Dálya	BIH
2142	*H. abietinum*	*Abies alba*	stump	43.349231	18.613256	14.11.2022	L. B. Dálya	BIH
2143	*H. annosum*	*Pinus sylvestris*	log	44.130289	17.371765	18.11.2022	L. B. Dálya	BIH
2144	*H. abietinum*	*Picea abies*	stump	44.143238	17.360008	18.11.2022	L. B. Dálya	BIH
2145	*H. abietinum*	*Abies alba*	stump	44.145277	17.373360	18.11.2022	L. B. Dálya	BIH
2146	*H. abietinum*	*Abies alba*	stump	44.144223	17.377767	18.11.2022	L. B. Dálya	BIH

[a] On which the fruiting body used for isolation was growing. [b] RNA-Seq was performed for the single isolate.

Table S2. Types and genomic position of the ribozymes detected in *Heterobasidion* ambiviruses.

Virus acronym	Rbz type	Initial genomic position[a]	Final genomic position[a]	Rbz polarity	E value	Rbz sequence
HetAlV1	HPRz	358	257	−	2.3e−09	complete
HetAlV1	HPRz	4657	4754	+	4.6e−08	partial
HetAlV30	HHRz	1	52	+	5.8e−11	complete
HetAlV30	HPRz	396	304	−	3.3e−11	complete
HetAlV31	HHRz	1	51	+	3.8e−09	partial
HetAlV31	HPRz	352	283	−	2.5e−08	complete
HetAlV32	HPRz	4350	4450	+	4.4e−09	partial
HetAlV34	HHRz	4640	4691	+	1.3e−11	complete
HetAlV34	HPRz	4956	4923	−	0.11	partial
HetAlV35	HPRz	163	86	−	1.9e−11	complete
HetAlV36	HPRz	71	8	−	2.3e−09	complete
HetAlV36	HHRz	4607	4658	+	1.9e−12	complete
HetAlV37	HHRz	4718	4767	+	2.5e−07	partial
HetAlV38	HHRz	4713	4762	+	2.5e−07	partial
HetAlV39	HPRz	66	4	−	1e−09	complete
HetAlV40	HHRz	2050	2101	+	3e−12	complete
HetAlV40	HPRz	2405	2343	−	1.2e−09	complete

[a] nt positions of the region spanning the ribozyme. The (+) polarity is defined as the RNA strand coding for the polymerase (ORFA).

Table S3. Short contigs resembling ambi-like viruses detected in RNA-Seq dataset BIH.

Contig name	Length (nt)	% GC	Mapped reads[a]	Mean depth	BLASTX first hit by max score[b]	Identity	Query cover	E value
NODE_26	3518	45.7%	15,192	580	Heterobasidion ambi-like virus 6 (UOX39308)	77.8%	63%	0
NODE_44	2503	51.5%	55,629	3009	Heterobasidion ambi-like virus 3 (WOK44140)	65.9%	70%	0
NODE_52	2250	49.4%	25,282	1516	Heterobasidion ambi-like virus 10 (WOK44142)	68.6%	87%	0
NODE_55	2192	47.5%	39,634	2072	Heterobasidion ambi-like virus 12 (WOK44145)	95.9%	94%	0
NODE_56	2184	49.2%	18,332	1096	Heterobasidion ambi-like virus 10 (WOK44142)	69.5%	89%	0
NODE_58	2111	49.5%	16,019	921	Heterobasidion ambi-like virus 10 (WOK44142)	69.2%	92%	0
NODE_59	2101	45.1%	12,613	750	Heterobasidion ambi-like virus 15 (WNH24528)	76.4%	99%	0
NODE_68	2003	51.4%	38,992	2474	Heterobasidion ambi-like virus 3 (UOX39303)	65.1%	95%	0
NODE_70	1982	51.2%	40,501	2635	Heterobasidion ambi-like virus 3 (UOX39303)	64.6%	95%	0
NODE_72	1929	50.1%	52,969	3675	Heterobasidion ambi-like virus 3 (UHK02576)	67.5%	92%	0
NODE_73	1903	52.2%	3401	239	Heterobasidion ambi-like virus 15 (WNH24527)	47.1%	95%	9e−175
NODE_74	1901	48.8%	47,459	3243	Heterobasidion ambi-like virus 3 (UHK02576)	68.8%	99%	0
NODE_78	1840	48.3%	4658	265	Heterobasidion ambi-like virus 6 (UOX39308)	60.7%	99%	0
NODE_80	1793	47.7%	527,396	39,990	Heterobasidion ambi-like virus 1 (UHK02572)	97.2%	96%	0
NODE_82	1743	47.2%	15,124	1165	Heterobasidion ambi-like virus 10 (WOK44143)	73.4%	92%	0
NODE_87	1665	49.6%	374,543	30,871	Heterobasidion ambi-like virus 1 (UHK02573)	97.1%	56%	0
NODE_90	1564	49.4%	324,488	24,300	Heterobasidion ambi-like virus 1 (UHK02573)	94.9%	41%	1e−143

NODE_110	1389	49.6%	4032	370	Heterobasidion ambi-like virus 15 (WNH24527)	68.2%	99%	0
NODE_116	1363	49.9%	319,531	31,138	Heterobasidion ambi-like virus 1 (UHK02573)	94.4%	47%	7e−144
NODE_125	1315	49.6%	22,155	2347	Heterobasidion ambi-like virus 12 (WOK44144)	97.5%	99%	0
NODE_157	1133	47.5%	3855	420	Heterobasidion ambi-like virus 15 (WNH24527)	73.5%	99%	0
NODE_169	1080	51.2%	6875	823	Heterobasidion ambi-like virus 15 (WNH24527)	52.9%	89%	1e−100
NODE_172	1072	48.7%	304,446	37,390	Heterobasidion ambi-like virus 1 (UHK02572)	91.1%	94%	0
NODE_195	1008	47.7%	7850	933	Heterobasidion ambi-like virus 10 (WOK44143)	81.1%	99%	0
NODE_196	1000	48.1%	13,133	1306	Heterobasidion ambi-like virus 10 (WOK44143)	62.4%	66%	2e−92
NODE_197	1000	49.3%	31,709	3941	Heterobasidion ambi-like virus 3 (UHK02576)	63.6%	99%	3e−135
NODE_200	980	50.5%	1770	242	Heterobasidion ambi-like virus 10 (WOK44142)	72.1%	99%	3e−165
NODE_212	929	48.9%	15,668	1500	Heterobasidion ambi-like virus 4 (UHK02578)	80.3%	99%	0
NODE_247	811	47.3%	14,711	2187	Heterobasidion ambi-like virus 10 (WOK44143)	83.9%	41%	1e−23
NODE_255	790	49.1%	13,238	1963	Heterobasidion ambi-like virus 27 (WIW43252)	72.1%	99%	1e−132
NODE_260	784	49.0%	5686	1012	Heterobasidion ambi-like virus 10 (WOK44143)	82.4%	99%	3e−156
NODE_265	774	52.5%	19,392	3049	Heterobasidion ambi-like virus 3 (UOX39303)	60.5%	99%	5e−106
NODE_279	730	47.0%	555,986	76,461	Heterobasidion ambi-like virus 6 (UOX39308)	53.7%	92%	3e−66
NODE_297	692	50.4%	13,397	2522	Heterobasidion ambi-like virus 27 (WIW43251)	70.6%	36%	7e−24
NODE_414	554	48.2%	1379	334	Heterobasidion ambi-like virus 15 (WNH24528)	66.8%	99%	2e−79
NODE_422	546	47.6%	17,641	3615	Heterobasidion ambi-like virus 4 (UHK02578)	80.9%	95%	9e−94

NODE_449	531	46.3%	4000	835	Heterobasidion ambi-like virus 10 (WOK44143)	81.0%	98%	7e−97

[a] Using Geneious assembler with medium-low sensitivity.
[b] Search done on 18.05.2024.

Table S4. Viruses included in the DAPC analysis.

Mycovirus	Host species	Host strain origin	GenBank ID
Heterobasidion ambi-like virus 1	*H. parviporum*	Finland	MZ502384
Heterobasidion ambi-like virus 1	*H. abietinum*	Bosnia	PP744446*
Heterobasidion ambi-like virus 2	*H. parviporum*	Finland	MZ502385
Heterobasidion ambi-like virus 2	*H. abietinum* OR *H. annosum*	Bosnia	PP744447*
Heterobasidion ambi-like virus 3	*H. parviporum*	Finland	MZ502386
Heterobasidion ambi-like virus 3	*H. parviporum*	Finland	OR607751
Heterobasidion ambi-like virus 3	*H. parviporum*	Finland	OR607752
Heterobasidion ambi-like virus 3	*H. parviporum*	Finland	OR644497
Heterobasidion ambi-like virus 3	*H. parviporum*	Czechia	ON014526
Heterobasidion ambi-like virus 4	*H. parviporum*	Finland	MZ502387
Heterobasidion ambi-like virus 5	*H. annosum*	Czechia	ON014527
Heterobasidion ambi-like virus 6	*H. annosum*	Czechia	ON014528
Heterobasidion ambi-like virus 7	*H. annosum*	Czechia	ON014529
Heterobasidion ambi-like virus 8	*H. annosum*	Czechia	ON014530
Heterobasidion ambi-like virus 9	*H. annosum*	Czechia	ON014531
Heterobasidion ambi-like virus 10	*H. parviporum*	Finland	OR644498
Heterobasidion ambi-like virus 12	*H. parviporum*	Finland	OR644499
Heterobasidion ambi-like virus 13	*H. parviporum*	Finland	OR644500
Heterobasidion ambi-like virus 14	*H. annosum*	Finland	OR343733
Heterobasidion ambi-like virus 14	*H. annosum*	Finland	OR343734
Heterobasidion ambi-like virus 15	*H. annosum*	Finland	OR343735
Heterobasidion ambi-like virus 19	*H. annosum*	Czechia	ON985229
Heterobasidion ambi-like virus 20	*H. annosum*	Czechia	ON985230
Heterobasidion ambi-like virus 21	*H. annosum*	Czechia	ON985231
Heterobasidion ambi-like virus 22	*H. annosum*	Czechia	ON985232
Heterobasidion ambi-like virus 23	*H. annosum*	Czechia	ON985233
Heterobasidion ambi-like virus 24	*H. annosum*	Czechia	OR031238
Heterobasidion ambi-like virus 25	*H. annosum*	Czechia	OR031239
Heterobasidion ambi-like virus 26	*H. annosum*	Czechia	OR031240
Heterobasidion ambi-like virus 27	*H. annosum*	Czechia	OR031241
Heterobasidion ambi-like virus 29	*H. annosum*	Czechia	OR031243
Heterobasidion ambi-like virus 30	*H. abietinum*	Bosnia	PP744448*
Heterobasidion ambi-like virus 31	*H. abietinum*	Bosnia	PP744449*
Heterobasidion ambi-like virus 31	*H. abietinum*	Bosnia	n.a.*
Heterobasidion ambi-like virus 32	*H. abietinum*	Bosnia	PP744450*
Heterobasidion ambi-like virus 33	*H. abietinum*	Bosnia	PP744451*
Heterobasidion ambi-like virus 34	*H. abietinum* OR *H. annosum*	Bosnia	PP744452*
Heterobasidion ambi-like virus 35	*H. abietinum* OR *H. annosum*	Bosnia	PP744453*
Heterobasidion ambi-like virus 36	*H. abietinum* OR *H. annosum*	Bosnia	PP744454*
Heterobasidion ambi-like virus 37	*H. abietinum* OR *H. annosum*	Bosnia	PP744455*
Heterobasidion ambi-like virus 38	*H. abietinum* OR *H. annosum*	Bosnia	PP744456*
Heterobasidion ambi-like virus 39	*H. abietinum* OR *H. annosum*	Bosnia	PP744457*
Heterobasidion ambi-like virus 40	*H. abietinum* OR *H. annosum*	Bosnia	PP744458*

*Reported in this study; n.a., not available.
